# Investigation of the Erosion Damage Mechanism and Erosion Prediction of Boronized Coatings at Elevated Temperatures

**DOI:** 10.3390/ma14010123

**Published:** 2020-12-30

**Authors:** Liu-Xi Cai, Yun Li, Shun-Sen Wang, Yao He, Fang Li, Ze-Kun Liu

**Affiliations:** 1 School of Chemical Engineering and Technology, Xi’an Jiaotong University, Xi’an 710049, China; liuxicai@xjtu.edu.cn (L.-X.C.); yunli@mail.xjtu.edu.cn (Y.L.); heyoox163@163.com (Y.H.); a772601806@stu.xjtu.edu.cn (Z.-K.L.); 2 State Key Laboratory of Multiphase Flow in Power Engineering, Xi’an Jiaotong University, Xi’an 710049, China; leyoushijie@163.com

**Keywords:** boronized coating, erosion damage mechanism, high-temperature erosion experiment, particle size, erosion prediction

## Abstract

In this study, the high temperature erosion mechanisms and damage characteristics of a boronized coating have been systematically studied by employing an improved high-temperature accelerated erosion test bench and impact contact theory analyses. Within the scope of the experimental parameters, the erosion rate of the boronized coating under the same erosion conditions was observed to be only one half to one-twelfth of the erosion rate of the substrate. Furthermore, the boronized coating was noted to be less sensitive to the speed of the erosion particles than the plastic substrate, thus, indicating superior and more stable erosion resistance than the base material. The boronized coating exhibited typical brittle fracture characteristics under impact by the high-speed particles. When the particle impact normal stress exceeded the critical stress for crack propagation owing to the coating defects, the surface and subsurface layers of the coating initially formed horizontal and vertical micro-cracks, followed by their gradual expansion and intersection. After destabilization, the brittle coating material was peeled layer-by-layer from the surface of the test piece. At the same incident speed, as the particle size was increased from 65 μm to 226 μm and 336 μm, the size (width) of the erosion cracks on the coating surface increased from 1 μm to 30 μm and 100 μm respectively. Correspondingly, the erosion damage thickness of the coating was enhanced from 15 μm to 50 μm and 100 μm. In the case of the quartz sand particle size exceeding 300 μm, the dual-phase boronized coating did not provide effective protection to the substrate. Furthermore, based on the elastoplastic fracture theory, a prediction model for the erosion weight loss of the boronized coatings within the effective thickness range has been proposed in this study.

## 1. Introduction

Erosion caused by the solid particles impacting at high speed is commonly observed in industrial production and is one of the main factors impacting the equipment operation safety and economy negatively [1,2]. In thermal power plants, the erosion caused by fly ash and coal dust on the draught fan blades of the boiler may reduce the total pressure by more than 60% [3], and the oxide particles entering the steam turbine with the steam further erode the key components of the equipment, such as main valve, regulating valve and blade cascades. It leads to the valve control failure and reduced service life of the blade cascades, thereby, significantly reducing the economic advantage resulting due to the increment in the initial parameters of the unit [4]. In the processes of pneumatic as well as oil and gas transportation, the erosion damage by particles to the elbow reduces its life to only 1/50 of the straight pipe section [5]. On the other hand, the erosion by sand particles on the blades of turbo compressors in a dusty environment causes the compressor performance to drop sharply, leading to eventual shut down [6]. Numerous similar cases are available, impacting the national economic development owing to the significant economic losses. With the development of equipment with high capacity, harsh working conditions and extensive integration, the extent of damage becomes even higher.

At present, the commonly used method to overcome the particle erosion issue is to coat the surface of the material with a hard coating so as to improve its passive anti-wear performance. Two main types of strengthening coatings are commonly used. In the first case, interstitial solid solutions are formed on a metal surface through atomic diffusion, such as the thermal spray coating [7]. In other case, new compounds are formed on the surface through the reaction of the diffused atoms and base metal, such as boronized coatings [8]. Compared with the thermal spray coating, the thermal diffusion boronized coating exhibits compact structure and higher coating hardness, along with superior erosion resistance [9,10]. Therefore, the boronized coating is widely used in the industrial processes for strengthening of the substrates.

With respect to the anti-wear performance of the boronized coating, Biddulph et al. [11] summarized that the boronized coating exhibited excellent resistance to sliding and erosion wear, and extended the life of the mold by more than 10 times. Based on the disc wear test, Tabur et al. [12] pointed out that, compared with the base material AISI8620 steel, the boronized coating enhanced the wear resistance of the material by five times. Saduman et al. [13] conducted a low-speed (1 m/s) sliding wear test using a similar test bench and observed that the wear rate of the boronized coating was only 10% of that of the AISI4140 substrate. The dry sliding wear test conducted by Taktak [14] revealed that, at 600 ℃, the wear resistance of the boronized coating was 3 and 2.5 times that of the base materials 52100 steel and 440C steel, respectively. Medvedovski et al. [15] simulated the wear behavior of the oil mining equipment components using the reciprocating sliding wear test bench. It was observed that, due to the high hardness and bonding strength of the dual-phase boronized coating, its wear-corrosion resistance was 30 times that of the carbon steel and other coatings (e.g., electroplated nickel coatings). In fact, the boronized coating was significantly effective for the low alloy steels, chromium-molybdenum-based alloy steels and cobalt-based alloys, improving the wear resistance of the substrates by four to five times [16].

To study the anti-erosion performance of the boronized coating, Qureshi et al. [17] used 75 μm chromite particles as the erosion particles with impact velocities of 228~304 m/s and impact angles of 22.5° and 90°. The erosion rate of the boronized coating after process optimization was only one-third of that of the substrate 403SS. B.S. Mann [18] employed a rotating disc to model the erosion environment of the steam turbine blades at room temperature. It was reported that the erosion resistance of the boronized coating was 7.5 to 9.2 times that of the blade substrate (12Cr steel). By employing systematic high-temperature erosion tests, Wang et al. [19] concluded that the collapse erosion occurred in a 10 μm-thick boronized coating when the erosion particles were at diverse attacking angles. On the other hand, the erosion took place in a 20 μm-thick boronized coating when the erosion particles were at 75° and 90° attack angles. The coating was noted to swiftly fall off locally and fail. In another study, Cai et al. [20] conducted high-temperature erosion tests on three typical boronized coatings and observed that the single-phase Fe_2_B coatings exhibited a superior erosion resistance to that of the dual-phase boronized coatings. The numerical simulation results [21] further confirmed the experimental findings. Prescott [22] used 150 μm quartz sand particles to impact different boronized coatings and substrate test pieces at a speed of 36 m/s by employing the sandblasting test setup. It was reported that the erosion rate of the dual-phase boronized coating at a large erosion angle was equivalent to that of 316 stainless steel and ordinary carbon steel, however the erosion rate at a small angle was noted to be much smaller.

Although many studies have reported the wear and erosion performance of boronized coatings, the comparison of the erosion resistance (erosion rate) of the different substrates and coatings has been the main focus. The failure mechanisms of the coatings eroded by the hard particles have not been thoroughly studied. In addition, due to the lack of the high temperature erosion test data and numerical simulation results, the erosion rate prediction model for the boronized coatings under high temperature service environments has not been established. Such a model is vitally needed for the service life prediction and maintenance cycle formulation of boronized coatings. In this study, a systematic experimental investigation of the high-temperature particle erosion of the typical duplex phase boronized coatings was carried out by using an improved high-temperature accelerated erosion test bench. Combined with the microscopic and impact contact theory analyses, the erosion failure mechanism and influence of the erosion particle size on the erosion damage characteristics of the boronized coatings were revealed. Furthermore, based on the elastoplastic indentation and rupture theory, by analyzing and fitting the experimental data, a prediction model for the erosion rate of the boronized coatings was established. The findings from this study provide vital insights about the high temperature erosion damage mechanism of the brittle coatings, rational selection of the boronized coatings for different industrial applications and prediction of the coating service life.

## 2. Experimental Procedure

### 2.1. Test Facility Design and Measurements

**Figure 1:**
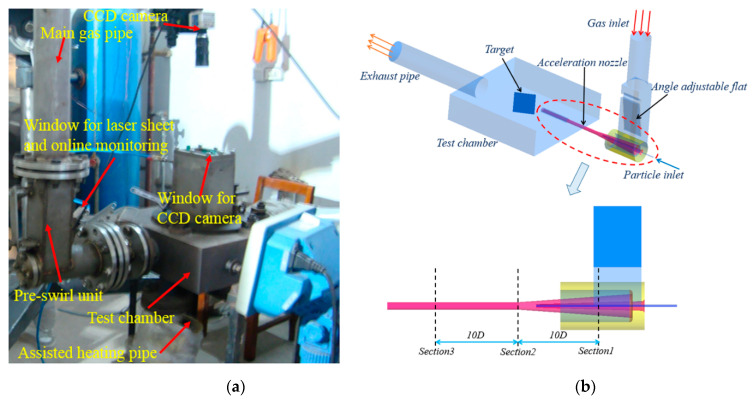
High-temperature accelerated erosion test system: (**a**) Image of the test system (**b**) 3D model of the test section.

Figure 1a is the physical diagram of the high-temperature accelerated erosion test system, whereas Figure 1b presents the three-dimensional model of the erosion test section. The working principle of the experimental system is as follows: gas from the combustion chamber is divided into two streams, one directly enters the test chamber to heat the specimen and establish a high-temperature erosion environment, whereas the other stream enters the acceleration nozzle to accelerate the solid particles. The solid particles from the screw feeder are directly transported by the high-pressure airflow to the throat of the acceleration nozzle. After being accelerated by the high-temperature gas, a uniform two-phase gas–solid mixture meeting the test requirements of temperature and speed is obtained. Subsequently, the solid particles hit the target material installed in the cavity of the test section at a certain inclination (*β*) to produce the corresponding erosion. The diameter of the main gas pipeline of the experimental system was 80 mm, the outlet diameter of the acceleration nozzle was 9 mm, and the diameter of the particle-conveying pipe at the outlet of the feeder was 4 mm.

**Figure 2:**
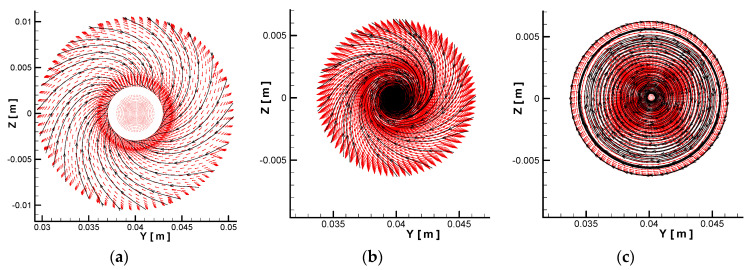
Evolution of the flow field structure at different cross-sections in the test system: (**a**) Section 1; (**b**) Section 2 (nozzle throat); (**c**) Section 3.

**Figure 3:**
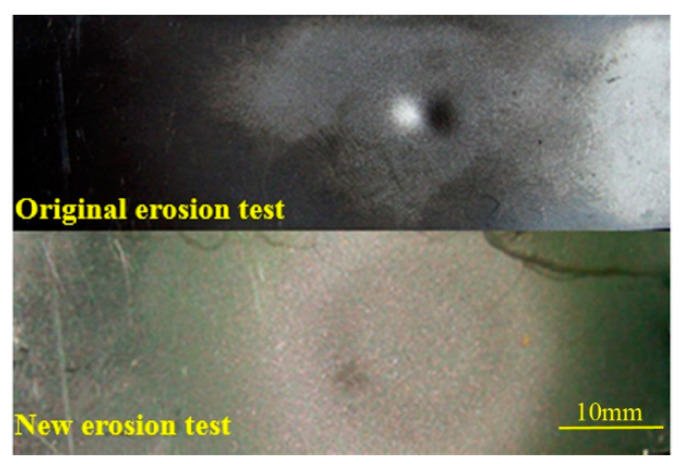
Comparison of the erosion scars on the target surface before and after the redesigning of the test sections.

In order to avoid the influence of the concentrated erosion by the particles in the accelerating nozzle on the measurement of the material erosion rate (as observed in the literature studies), an air flow pre-swirl device was installed before the accelerating nozzle, as shown in Figure 1. By adjusting the angle of the baffle, the main airflow produced a moderate swirl before entering the accelerating nozzle, as shown in Figure 2a. As the gas enters the accelerating nozzle and air velocity increases, the vortex dissipation is increased, with the vortex intensity increased correspondingly until the vortex intensity reaches the extreme value as the airflow reaches the nozzle throat, as shown in Figure 2b. Subsequently, the airflow enters the straight pipe section, and the factors that promote an enhancement in the vortex intensity are significantly weakened. On the other hand, the airflow velocity and vortex dissipation are still maintained at a high level, thus, the swirling intensity becomes weak, leading to the formation of a weak concentric swirl, as shown in Figure 2c. As the solid particles accelerate along the flow direction in the accelerating nozzle, the rotating airflow drags them to produce the radial diffusion. The rotating kinetic energy of the airflow is gradually dissipated on interaction with the wall and solid particles, and the spatial distribution of the particles becomes stable as the swirling flow dissipates. Finally, a uniform high-speed two-phase gas–solid jet is formed. Figure 3 shows the surface erosion morphology of the test piece before and after the improvement of the test section. It can be observed that the improved erosion test section was effective in avoiding the concentrated erosion (local pits) by the particles on the target surface, thus, improving the test accuracy.

In the high temperature erosion test, in order to verify the repeatability of the experimental findings under the same testing conditions, the experiments were performed on four targets in the same material by the rapid switching of the rotating mechanism. The erosion rate of the target can be obtained by measuring the cumulative mass *m_p_* of the particles involved in the erosion, as well as the mass *m*_1_ and *m*_2_ of the target before and after the erosion phenomenon. During the test, *m_p_* was measured by using an electronic balance, while *m*_1_ and *m*_2_ were determined by employing BS224S precision electronic balance from Sartorius, Germany, with a measurement accuracy of 0.1 mg.

**Figure 4:**
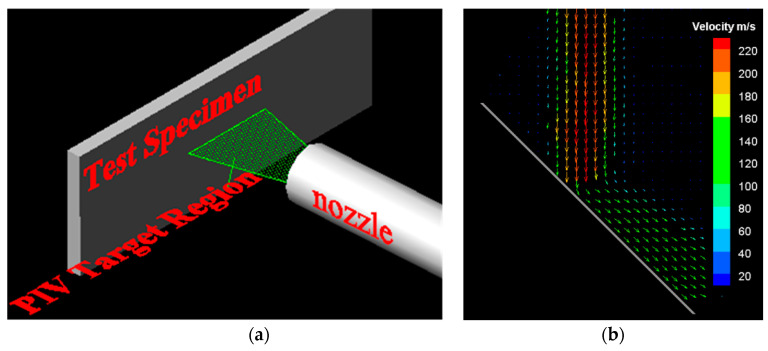
Schematic of the particle velocity field measurement by particle image velocimetry (PIV): (**a**) Test principle of PIV; (**b**) D32 = 65 μm under 210 m/s, 566 °C and 45 °C condition.

Particle image velocimetry (PIV) was used to measure the average velocity of the particles hitting the target surface, and the test principle has been presented in Figure 4a. The sheet light emitted by the Yttrium Aluminum Garnet (YAG) dual-pulse laser illuminates the flow field test area. In the direction perpendicular to the sheet light surface, an ImagePro4M CCD camera made from LaVision inc. of Germany is used to capture the moving images of the particles in the area illuminated by the sheet light. In the study, the YAG dual-pulse laser had a wavelength of 532 nm, and each pulse energy was 200 mJ. Furthermore, the pulse width and pulse frequency values were 5 ns and 15 Hz, respectively. The laser dual-pulse interval was adjusted from 0.5 μs to 0.1 ms, thus, realizing the measurement of the flow field from low to high speed. The ImagePro4M CCD image sensor had a pixel size of 2048 × 2048, with a sensing area of 15.1 mm × 15.1 mm and a maximum sampling rate of 16 fps. Using DiVis 8.0 software to perform the cross-correlation processing of the graphics, the particle velocity field was finally obtained, as shown in Figure 4b.

**Figure 5:**
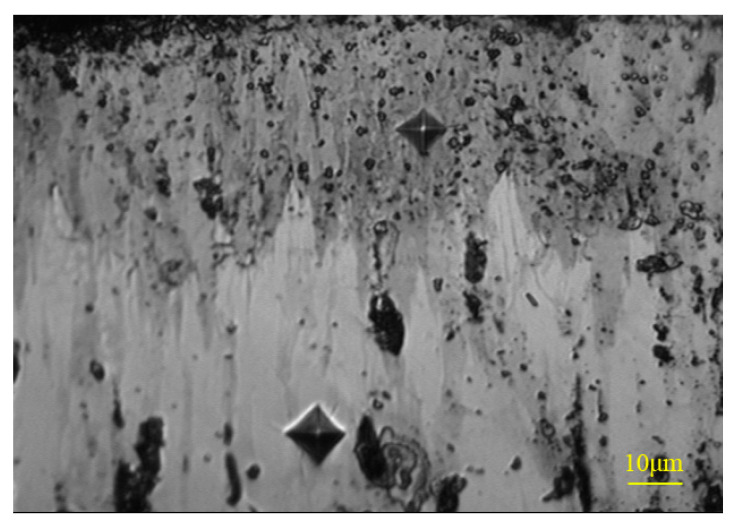
Coating hardness test.

A VEGAⅡ XUM scanning electron microscope (SEM) made from TESCAN inc. of Czech Republic was used to analyze the surface erosion morphology and coating thickness of the test piece. The average thickness of the coating was determined by taking the average of the thickness at 15 different locations. The composition of the boronized coating was confirmed by using SEM in the backscattering mode (BSE) and XRD analysis. A Buehler MICROMET 5104 automatic microhardness tester was used to measure the hardness of the polished cross-section of the boronized coating, at an applied load of 1 N, as shown in Figure 5. In addition, the Vickers hardness indentation method was used to measure the fracture toughness of the boronized coating, at an applied load of 3 N. The specific calculation procedure for the determination of the fracture toughness can be found in the literature [20]. In order to ascertain the reproducibility of the test data, the coating hardness and fracture toughness were reported after averaging 10 measurements under the same conditions.

### 2.2. Test Parameters and Conditions

**Figure 6:**
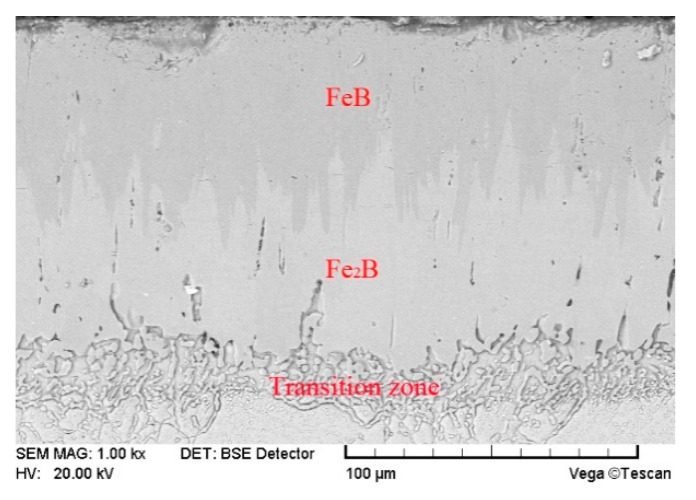
Cross-sectional morphology in backscattering (BSE) mode.

The base material of the test target was martensitic stainless steel 2Cr10MoVNbN. Its yield and tensile strength at room temperature were 860 MPa and 980 MPa, respectively. The elongation and reduction in area were 16% and 56%, respectively, with a hardness of 320 HB. The boronized coating was obtained by applying the solid boronizing powder to the stainless-steel substrate at a temperature of 1123~1273 K [20]. The detailed process is as follows: the substrate test piece was kept in the boronizing furnace for 10 h at a temperature of 1273 K, followed by cooling in air to room temperature. Finally, the tempering was carried out at 943 K for 8 h. As shown in Figure 6, in the backscattering mode, the prepared boronized coating exhibited a compact and typical dual-phase structure, with the top FeB phase and bottom Fe_2_B phase accounting for about 50% each and exhibiting the characteristics of the tooth-like growth. The microscopic analysis revealed that the total thickness of the boronized coating was 120 μm. Furthermore, the hardness and porosity of the coating were measured to be 1800 HV and <0.5%, respectively.

**Figure 7:**
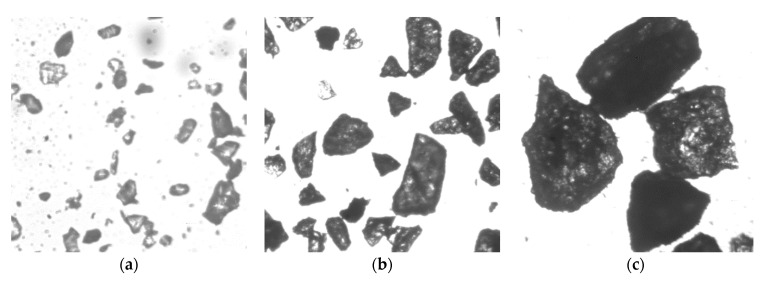
Microscopic morphology of the sand particles used in the test: (**a**) *D*_32_ = 65 μm; (**b**) *D*_32_ = 226 μm; (**c**) *D*_32_ = 336 μm.

**Figure 8:**
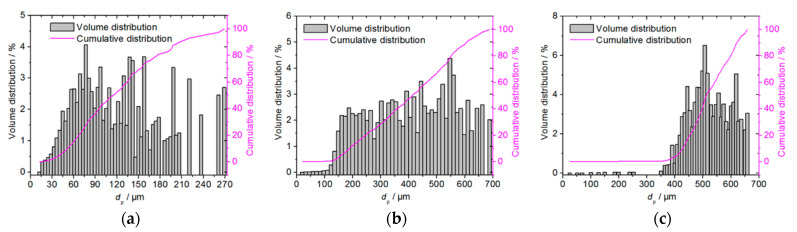
Size distribution of the sand particles used in the test: (**a**) *D*_32._ = 65 μm; (**b**) *D*_32_ = 226 μm; (**c**) *D*_32_ = 336 μm.

In the experiment, quartz sand particles were used as the erosion particles. The self-developed particle morphology and size analysis software was employed to analyze the size distribution of the erosion particles. The micro-morphology and particle size distribution of the quartz sand particles of different sizes are presented in Figure 7 and Figure 8. The quartz sand particles are noted to be angular, with the Sauter average diameters of the three kinds of particles as 65 μm, 226 μm and 336 μm.

Through systematic numerical simulation, the speed of the solid particles impacting the turbine blades was observed to be in the range 200 m/s~350 m/s [23], thus, two particle impact speeds of 210 m/s and 320 m/s were chosen. Based on the intake temperature of the supercritical steam turbine and final stage compression temperature of the aero-engine compressor, 566 °C was selected as the test temperature. For each sample, the coating erosion tests were conducted at seven particle incidence angles of 18°, 24°, 30°, 36°, 45°, 60°, 75° and 90°, keeping the same particle impact speed and air temperature.

## 3. Results and Discussion

### 3.1. Erosion Failure Mechanism of The Boronized Coating

**Figure 9:**
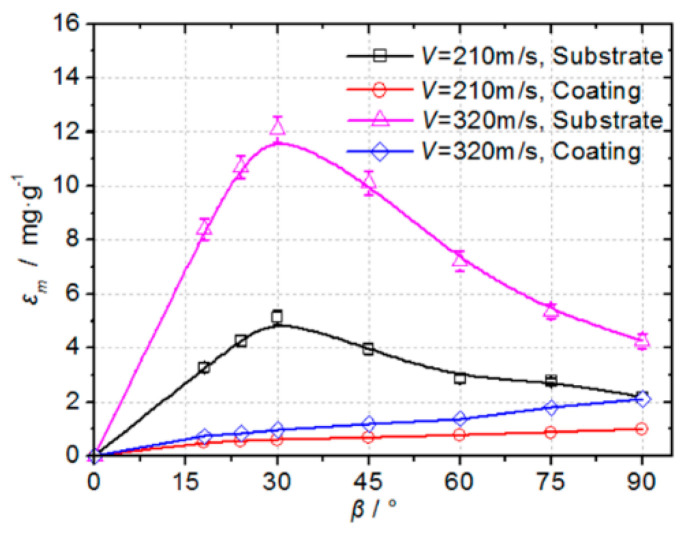
The variation of erosion rate with *β* for the boronized coating and substrate material (D_32_ = 226 μm).

Figure 9 shows the erosion rate of the 226 μm quartz sand particles at different speeds on the stainless-steel substrate and boronized coating. The erosion rate at each operating condition represents the average of four samples analyzed under the same operating conditions. It can be observed that at different incident velocities, the substrate exhibits obvious plastic erosion characteristics, and the maximum erosion rate occurs at an attack angle of 30°. On the other hand, the boronized coating exhibits brittle erosion characteristics, with the maximum erosion rate occurring at an attack angle of 90°. At an incident speed of 210 m/s, the erosion rates of the boronized coating at different angles are noted to be only one-half~one-eighth of the erosion rate of the substrate. Further, at the particle incident speed of 320 m/s, the erosion rates of the boronized coating at different angles are observed to be only one-half~one-twelfth of the erosion rate of the substrate. This indicates that, in the range of the particle speeds employed in the experiment, as the particle speed increases, the erosion rate of the boronized coating increases at a slower rate as compared to the substrate. In other words, the boronized coating is observed to be less sensitive to the particle speed than the plastic target, thus, exhibiting higher and stable erosion resistance than the base material.

**Figure 10:**
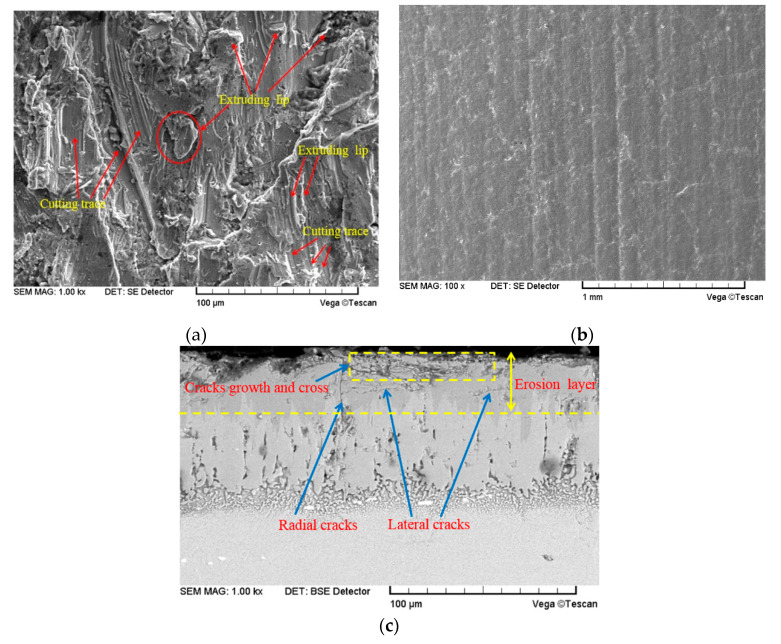
Erosion morphology of the target surface at 210 m/s, 566 ℃ and 30° condition (D_32_ = 226 μm): (**a**) Surface morphology of the substrate (**b**) Surface morphology of the boronized coating; (**c**) Cross-section morphology of the boronized coating.

Figure 10 shows the surface erosion morphology of the substrate and boronized coating after the erosion by the 226 μm quartz sand particles. As can be seen from Figure 10a, the surface of the substrate shows obvious traces of micro-cutting and furrowing. During the micro-cutting by the particle edges and corners, most of the traces on the plastic material were removed at once to form the wear debris. During furrowing and lip forming, the deformed lips on both sides and ends of the furrow were repeatedly deformed and fatigued owing to the multiple impacts, followed by falling off to form the wear debris. As can be seen from Figure 10b,c due to the superior hardness of the boronized coating, the erosion area did not exhibit large-scale plastic deformation. However, when the impact normal stress exceeded certain critical stress, the microcracks generated at the coating defects began to multiply and expand, thus, leading to the formation of the horizontal and longitudinal microcracks on the surface and subsurface of the coating. The cracks gradually expanded and intersected, and the surface of the specimen was peeled off layer-by-layer due to destabilization.

The comprehensive high-temperature erosion (Figure 9) and micro-erosion morphology (Figure 10) analyses reveal that the micro-cutting erosion rate of the quartz sand particles on the substrate at a small impact angle was much higher than that of the layer-by-layer peeling observed after crack propagation and coating destabilization. Therefore, for industrial applications where erosion happens by the particles at small impact angles, incorporation of the boronized coatings can effectively enhance the erosion resistance of the materials. On the other hand, at a large impact angle, due to the continuous enhancement of the particle impact normal stress at the same speed, the peeling rate of the unstable brittle coating materials due to the propagation of cracks in the coating increases, thus, the erosion rate of the boronized coating gradually increases. The observed variation of the erosion rate of the boronized coating as a function of the erosion angle is consistent with the literature reports [9,18,19,24]. The plastic substrate exhibited a lower rate of plastic deformation erosion under the impact of the particles at a large incident angle than that of micro-cutting and furrow erosion at a small angle, thus, the erosion rate gradually decreased as the incident angle increased beyond 30°.

In order to verify the erosion failure mechanism of the materials developed in this study, the critical stress value of the microcrack propagation at the defects in the boronized coating and impact normal stress value of the particles were estimated. It has been reported that the critical stress value *σ*_crit_ of the crack propagation at the defects (voids, stress concentration, etc.) in the coating material is related to the fracture toughness *K*_c_ of the material as follows [25,26]:

*σ*_crit = _*K*_c_ / (*πl*)^0.5^ (1)

where the initial defect length *l* is assumed between 0.1–1 μm.

For the dual-phase boronized coating investigated in this study, the results from the Vickers hardness analysis in reference [20] show that the fracture toughness of the FeB coating on the top is 1.5 MPa·m^1/2^, whereas the fracture toughness of the Fe_2_B coating on the bottom is 5.42 MPa·m^1/2^. From Equation (1), the critical stress value of the internal crack propagation for the FeB coating is calculated to be in the range 0.85 GPa~2.68 GPa, whereas the critical stress value of the internal crack propagation for the Fe_2_B coating is determined to be in the range 3.06 GPa~9.67 GPa.

The actual orientation of the angular quartz sand particles in contact with the wall surface of the coated target is difficult to be determined, thus, an equivalent spherical quartz sand particle of D_32_ = 226 μm impacting on the dual-phase boronized coating was taken as the research object in this study, and the transient impact stress of the spherical particle impacting normal to the coating surface was theoretically calculated.

According to the Hertz’s contact theory, the contact force F during the normal impact of a hard spherical particle on the surface of an infinitely large target can be expressed as [27]:

*F* = 1.036 *E*′ *D*^0.5^*h*^1.5^ (2)

where *D* is the particle diameter and *h* is the indentation depth. The effective elastic modulus *E*′ can be expressed as follows:

1/*E*′ = (1-*μ_p_^2^*)/ *E_p_* +(1-*μ_t_^2^*)/ *E_t_* (3)

where *E_p_* and *μ_p_* are the elastic modulus and Poisson’s ratio of the incident particle, respectively, whereas *E_t_* and *μ_t_* are the elastic modulus and Poisson’s ratio of the target, respectively.

The elastic potential energy during impact can be expressed as:

*E*_*e*_ = 0.5*Fh* (4)

At the moment the particle collides with the target surface, the kinetic energy of the particle is:

*E*_*P*_ = 0.5*mV*^2^ (5)

where *m* is the mass of the incident spherical particle, and *V* is the velocity of the particle hitting the target surface. Ignoring the energy loss during the impact, *E_e_* = *E_p_*. Thus, the maximum indentation depth of the particle can be obtained as:

*h*_max_ = (*mV*^2^/1.036 *E*′ *D*^0.5^)^0.4^ (6)

Similarly, ignoring the deformation and fragmentation of the particle during the impact, the contact area of the hard spherical particle impacting the target material (i.e., the area of the spherical cap pressed into the target material) is:

*S* = *πDh*_max_ (7)

The impact stress generated by particle hitting the target surface is:

*P* = *F*/*S* (8)

Incorporating Equations (2), (6) and 7 into Equation (8), the impact normal stress generated by the particles hitting the target surface at different speeds can be obtained. The material properties of the quartz sand particles and boronized coating used in the calculations are listed in Table 1. At the particle speed of 210 m/s, the stress generated by the spherical particles normal to the boronized coating is calculated to be 5.18 GPa, whereas the stress exceeds 6.13 GPa at the particle speed of 320 m/s. The observed normal stress of 5.18 GPa at the particle speed of 210 m/s is noted to exceed the upper limit of the critical crack propagation stress (2.68 GPa) for the FeB coating. Therefore, large-scale cracks are observed to appear on the surface of the coating at this condition. However, at the normal erosion at 320 m/s, due to the further increment in the impact normal stress, the coating surface appears to exhibit localized fragmentation. For the actual angular quartz sand particles, the contact area between the particle and coating wall at the moment of impact is smaller than the contact area between the equivalent spherical particle and coating surface. Therefore, the impact normal stress exerted by an angular quartz sand particle on the boronized coating is higher than the theoretically calculated value, which promotes the proliferation and propagation of microcracks in the boronized coating and correspondingly accelerates the erosion and spalling of the brittle coating materials.

### 3.2. Effect of Particle Size on the Erosion Damage of the Boronized Coating

**Figure 11:**
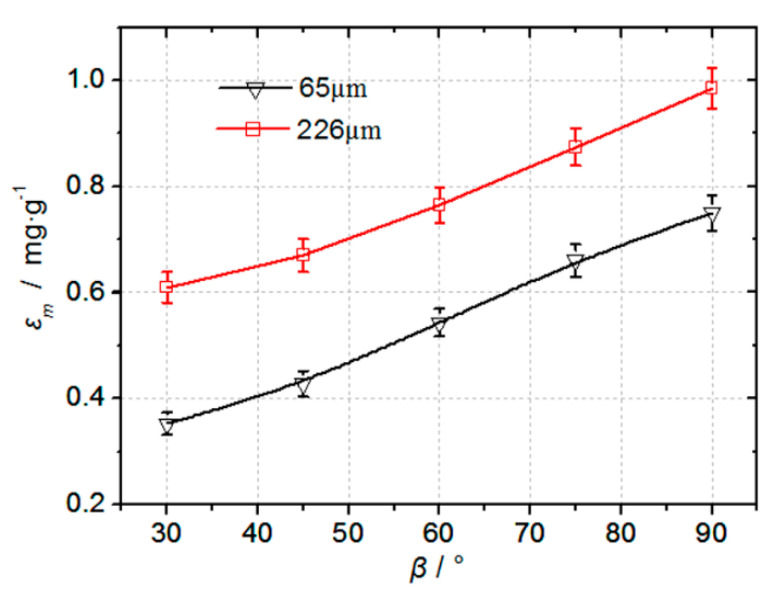
Comparison of the erosion rate curves for the quartz sand particles of 65 μm and 226 μm size at 210 m/s and 566 ℃.

Figure 11 shows the erosion rate curves of the quartz sand particles on the boronized coating at 210 m/s and 566 ℃. As the boronized coating swiftly fails and falls off after erosion by 336 μm particles, the erosion rate cannot be effectively measured, thus, the figure presents the erosion rate curves of 65 μm and 226 μm particles only. It can be observed that the shape of the erosion rate curves as a function of the incident angle for different particle sizes is very similar, thus, indicating that the erosion damage mechanism of the boronized coatings for different particle sizes is the same. At the same incident angle, as the particle size increases, the erosion rate of the coating is also enhanced. It is consistent with the experimental findings of Rutherford et al. [28] and numerical results of Hassani et al. [26]. Under the same erosion conditions, the erosion rate of 226 μm quartz sand particles is noted to be 1.31~1.73 times that of 65 μm particles.

**Figure 12:**
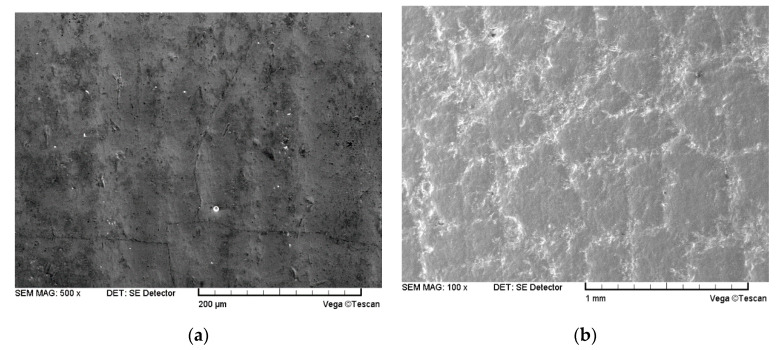
Comparison of the erosion morphology of the boronized coating at 210 m/s, 566 ℃ and 30° condition: (**a**) D_32_ = 65 μm; (**b**) D_32_ = 336 μm.

**Figure 13:**
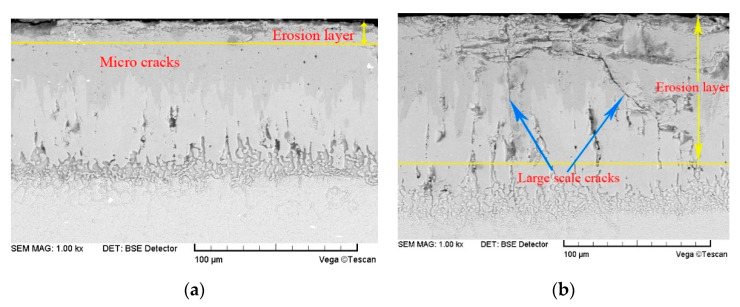
Erosion morphology of the cross-section at 210 m/s, 566 ℃ and 30° condition: (**a**) D_32_ = 65 μm; (**b**) D_32_ = 336 μm.

**Figure 14:**
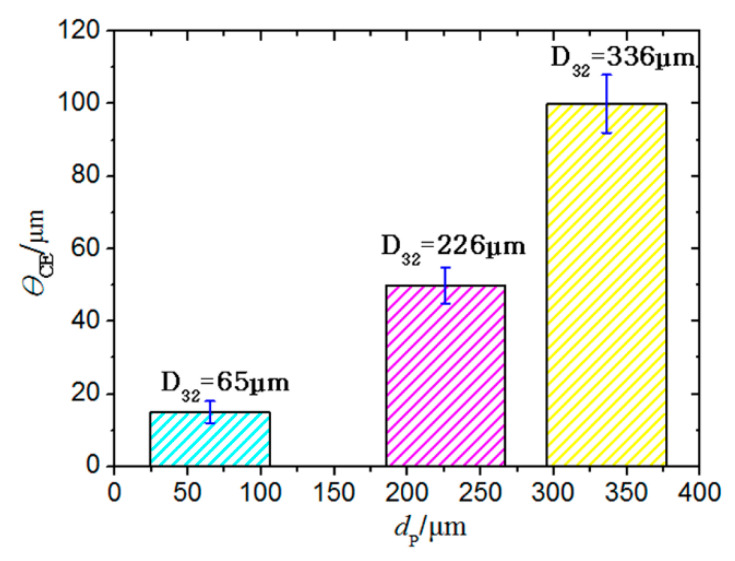
Thickness of the erosion layer as a function of the diameter of the erodent particles.

Figure 12 shows the micro-morphology of the coating surface under erosion by the particles of different sizes, whereas Figure 13 presents the cross-sectional crack morphology of the coating. Under the accelerated erosion test conditions, the erosion damage layer thickness in the boronized coating varies with the particle size, as shown in Figure 14. Under continuous impact of 65 μm particles, the microcracks with a width of about 1 μm appear on the surface of the boronized coating, and the thickness of the particle erosion damage layer in the cross-sectional direction is in the range of 15 μm. However, in the case of erosion by high-speed particles of 226 μm in size, the crack size on the surface of the boronized coating increases to 20–30 μm, whereas the thickness of the particle erosion damage layer in the cross-sectional direction reaches 50 μm. As the particle size is further increased to 336 μm, the size of the cracks on the coating surface reaches 50–100 μm after 5 s (about 10 g), and the thickness of the particle erosion damage layer at the cross-section reaches 100 μm. After 10 s (about 20 g), the coating is noted to be completely broken. Overall, increasing the particle size exponentially enhances the impact force of the particles at the same impact velocity [26]. As the size of the cracks on the surface and inside the coating increases, the rate of expansion and destabilization also increases sharply, thus, making coating more likely to peel off in large pieces, leading to breakage and failure. The authors’ findings from the actual service process of the boronized coating also confirm the observed phenomenon [29].

**Figure 15:**
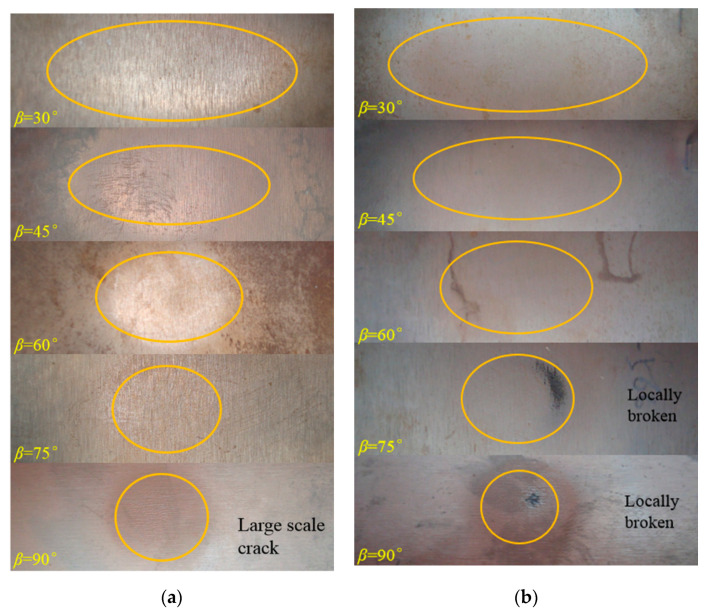
Macroscopic erosion morphology of the boronized coating at 210 m/s and 566 ℃ (D32 = 65 μm): (**a**) 210 m/s; (**b**) 320 m/s.

**Figure 16:**
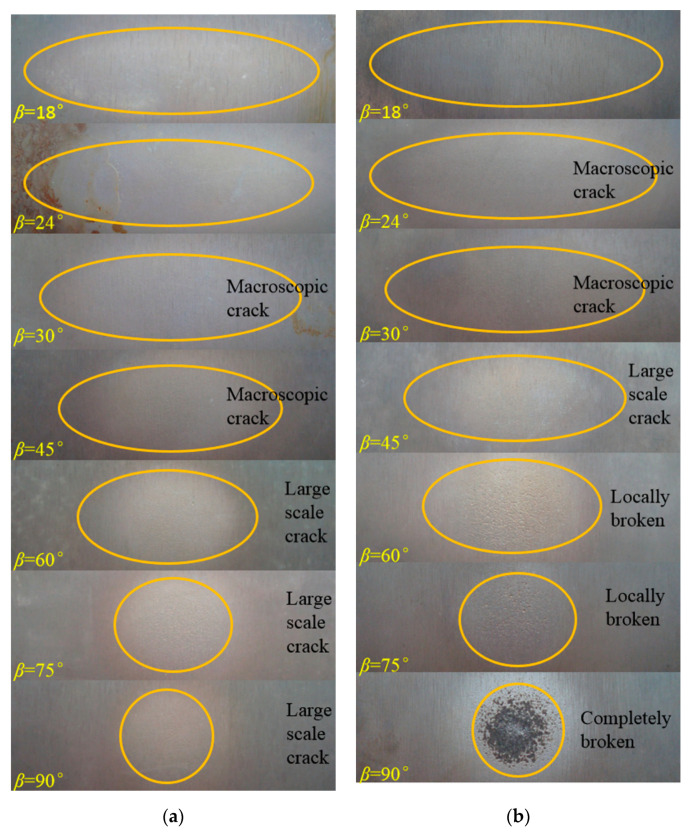
Macroscopic erosion morphology of the boronized coating at 210 m/s and 566 ℃ (D32 = 226 μm): (**a**) 210 m/s; (**b**) 320 m/s.

Figure 15 and Figure 16 further present the macroscopic erosion morphology of the surface of the boronized coating impacted with the quartz sand particles of sizes D_32_ = 65 μm and D_32_ = 226 μm. For the same particle size, as the impact angle increases, the shape of the erosion spots on the surface of the specimen gradually transform from ellipses to a circle. At the same time, due to an increase in the normal impact stress, the size of the cracks in the erosion area gradually increase, causing the coating to eventually break off the surface.

At the same impact speed, as the size of the erosion particles increases, the incident angle of the particles leading to the macroscopic cracks on the coating surface advances. At 210 m/s, no macro cracks were observed in the erosion area for 65 μm particles at an attack angle of 75°. On the other hand, the macro cracks were noted to appear on the coating surface in the case of impact with 226 μm particles at an angle of 30°, which further evolved into large-scale cracks at an attack angle of 60°. As the particle size increased to 336 μm, under the erosion conditions of 210 m/s and 30°, it only took 10 s for the coating to be completely eroded and broken. Combining with the authors’ earlier findings [19], because the brittle material has a critical coating thickness corresponding to the eggshell effect, thus, for the coating thickness less than the critical thickness, any particle impacting the coating causes the coating to partially fall off. In the case of erosion by the high-speed 40 μm iron oxide particles, the critical thickness of the boronized coating has been reported to be about 20 μm [19]. The experimental findings in this study exhibit that the critical thickness of the erosion failure of the boronized coating increases with the particle size. Additionally, the effective thickness during the service life decreases, thus, shortening the service life accordingly. Therefore, in practical applications, the service life of the coating within the effective thickness range is meaningful, and filtering the large particles in the airflow is extremely vital to retain the service life of the protective coating on the components of the power equipment.

### 3.3. Model to Predict The Erosion Rate of The Boronized Coating

At present, the elastoplastic indentation fracture theory proposed by Evans et al. [30] is commonly employed to analyze the erosion and wear of brittle material. The theory assumes that the erosion failure of the brittle targets is dominated by the brittle fracture mechanism, and the particles do not break during the impact (rigid particles). Based on this, the theory assumes that the volume of the material eroded by a single particle is approximately equal to the volume of a cylinder whose radius is the maximum radial crack length and height is the maximum horizontal crack depth. Furthermore, it is assumed that the material loss is approximately the sum of the material removed by a single impact of the particles that do not affect each other. Evans et al. derived the volume expression of the solid particles eroding the brittle targets based on the elastoplastic theory as follows:

*E_V_* ≈*k V*^*e*1^*R_p_*^*e*2^*ρ_p_*^*e*3^*K_c_*^*e*4^*H*^*e*5^ (9)

where *E_V_* is the erosion volume of the brittle target, *k* is a dimensionless constant, *V* is the particle impact velocity, *R_p_* is the incident particle diameter, *ρ_p_* is the particle density, *K_c_* is the fracture toughness of the brittle target, and *H* is the micro hardness of the target. *e*_1_, *e*_2_, *e*_3_, *e*_4_ and *e*_5_ are the indices determined experimentally, which show the relationship between *V*, *R_p_*, *ρ_p_*, *K_c_*, *H* and *E_V_*, respectively. The larger the index, the more sensitive the target erosion volume (*E_V_*) is to the parameter corresponding to the index.

Combining the findings from the high-temperature erosion experiments and microscopic analysis of the erosion morphology of the coating, it can be observed that the boronized coating exhibits an obvious brittle failure mechanism under the impact of the high-speed quartz sand particles. In addition, from the analysis presented in Section 3.2, the erosion process within the effective thickness of the boronized coating can be considered to be a steady state erosion process. It can also be noted from the macroscopic and microscopic erosion morphology that the erosion by the particles of size 65 μm and 226 μm within the effective thickness range of the boronized coating were not affected by the characteristics of the coating–substrate interface. The finite element simulation results of the particle erosion of the brittle coating by Bielawski et al. [31] and Hassani et al. [26] also confirm the observed phenomenon, i.e., the influence of the substrate and interface characteristics on the stable erosion process of the coating can be ignored when the coating thickness reaches a certain value. Therefore, the basic expression of elastoplastic theory can be used to establish a model to predict the erosion rate of the brittle coatings, which would be convenient for engineering applications.

The elastoplastic theory in Equation (9) proposes that the erosion volume of the brittle targets has an exponential relationship with the particle density. Hassani et al. [26] investigated it further by studying the relationship between the impact kinetic energy of the particles and erosion volume. The simulation results confirmed that the target erosion volume had a linear relationship with the particle density, i.e., *e*_2_ = 1. Aquaro et al. [32] derived the erosion volume of the brittle materials based on the Hertzian elasticity theory, which also exhibits approximately linear relation with the particle density. Therefore, considering the determined properties of the erosion particles and the target, a linear relationship between the erosion rate of the coating and particle density can be assumed.

In addition, because the elastoplastic theoretical model is based on the particles impacting the brittle target, the volume removal of the target shown in Equation (9) also represents the maximum volume of the material removed by a single particle during a single impact on the target. Considering that the erosion damage to the brittle materials by the particles is mainly due to their normal stress, it can be approximated that the volume of the target material removed during the inclined impact of the particles is proportional to the normal impact stress.

Based on the analysis and theoretical expression of the elastoplastic fracture of the brittle materials (Equation (9)), an expression for the erosive weight-loss per unit time of the boronized coating has been proposed as:

*E_m_* = *k q sinβ ρ_c_ ρ_p_ V ^a^ R_p_^b^ K_c_^c^ H ^d^* (10)

where *E_m_* is the erosive weight-loss of the boronized coating per unit time, *q* is the mass of the incoming particles per unit time, and *β* is the incident angle of the particles. Furthermore, *a*, *b*, *c* and *d* are the experimentally determined exponents, and the other parameters are the same as in Equation (9).

Based on the erosion results of the boronized coating under the impact of quartz sand particles in this study and the erosion test data of different boronized coatings under the impact of 45 μm iron oxide particles in literature [20], a prediction relationship for the erosive weight-loss of the boronized coating per unit time was established by using the multiple linear regression method:

*E_m_* = 4.92*q sinβ ρ_c_ ρ_p_* (*V/100*)^2.35^*R_p_*^1.26^/( *K_c_*^1.64^*H*^1.08^) (11)

where *E_m_* is the erosive weight-loss of the boronized coating per unit time (mg·h^−1^), *q* is the mass of the incoming particles per unit time (kg·h^−1^), *β* is the incident angle of the particles (°), *ρ_c_* is the coating density (g·cm^−3^), *ρ_p_* is the particle density (g·cm^−3^), *V* is the particle impact velocity (m·s^−1^), *R_p_* is the incident particle diameter (μm), *K_c_* is the fracture toughness of the brittle target (MPa∙m^1/2^), and *H* is the microhardness of the target (GPa).

In order to investigate the reliability of the developed model, the experimental data not included during fitting were substituted in Equation (11) to predict the erosion values for comparison with the experimental findings. It is noted that the error between the predicted and experimental values for the impact angles of 75° and 90° was significant (about 11.8%). On the other hand, the error between the predicted and experimental values for other impact angles was observed within 6%, thus, verifying the reliability of the proposed erosion prediction model for the boronized coating.

Table 2 further displays the comparison of the exponents in different erosion predictive models for brittle material. *e*_1_, *e*_2_, *e*_3_, *e*_4_, and *e*_5_ are the exponents for particle impact velocity, particle diameter, particle density, fracture toughness of the target and micro hardness of the target, respectively. As we can see, the exponents for particle impact velocity *e*_1_, particle density *e*_3_, and target fracture toughness *e*_4_ in different erosion predictive models are in a concentrated range of 2.35~3.2, 1.0~1.58 and −1.3~−1.64, respectively, while the exponents for particle diameter *e*_2_ and target hardness *e*_5_ are in a scattered range. The difference among these four models is mainly caused by the research methods adopted by different researchers, the properties of the particles and brittle target materials, and the erosion parameters among different research.

In specific applications, when the operating conditions of the equipment are known, the properties of the boronized coating, such as *ρ_c_*, *K_c_* and *H*, can be obtained from the material datasheets or through the material tests. *q*, *R_p_*, and other particle parameters can be obtained through bypass, particle pre-separation devices, online monitoring, etc. The velocity *V* and angle *β* of the particles impacting the wall of the flow channel can be obtained by numerical simulation. Therefore, Equation (11) can be used to accurately calculate the weight loss per unit time of the strengthening coating, thus, allowing the estimation of the effective service life of the coating.

## 4. Conclusions

Studying the mechanism of the high temperature erosion damage of coatings is of vital significance to minimize the damages to power equipment components by solid particles. In this study, the high temperature erosion mechanism and damage characteristics of a thermal diffusion boronized coating have been systematically studied by using an improved high-temperature accelerated erosion test bench. Combined with the microscopic performance and impact contact theory, the erosion failure mechanism and influence of the particle size on the erosion damage characteristics of the boronized coatings have been revealed. Furthermore, based on the theory of the elastoplastic indentation and rupture, a model to predict the erosion rate of the boronized coating has been established for the engineering applications. The following main conclusions can be drawn:

1. For the experimental conditions used in the study, the erosion rate of the boronized coating at the same erosion conditions was noted to be only one-half~one-twelfth of the erosion rate of the substrate, and the boronized coating was observed to be less sensitive to the speed of the erosion particles than the plastic substrate. For industrial applications involving small-angle particle erosion as the main cause of the erosion failure, effective use of the boronized strengthening coating can effectively enhance the erosion resistance and service life of the material.
2. High temperature erosion analysis, erosion morphology microanalysis and collision contact theory indicate that the boronized coating exhibits typical brittle fracture characteristics on impact by the high-speed particles. When the particle impact normal stress exceeds the critical stress for crack propagation in the coating defects, the surface and subsurface layers of the coating initially form the horizontal and vertical micro-cracks, leading to the gradual expansion and intersection. After destabilization, the brittle coating material is peeled off layer-by-layer from the surface of the test piece.
3. At the same incident speed, as the particle size increases from 65 μm to 226 μm and 336 μm, the size (width) of the erosion cracks on the coating surface is noted to increase from 1 μm to 30 μm and 100 μm, respectively. Correspondingly, the erosion damage thickness of the coating also increases from 15 μm to 50 μm and 100 μm. In practical applications, it is vital to effectively filter the large size particles in the airflow to extend the service life of boronized coatings.
4. Based on the elastoplastic fracture theory of the brittle materials, a prediction model has been proposed in this study for the erosion weight loss of the boronized coatings within the effective thickness range, considering the influence of the incident angle on the erosion rate of the brittle materials. It also provides a reference method for the establishment of the particle erosion life prediction models for other coating types.

The findings from this study contribute towards the understanding of the high temperature erosion damage mechanism of the brittle coatings, rational selection of the boronized coatings in diverse industrial applications and prediction of the coating service life.

## Figures and Tables

**Table 1:** Material parameters of the quartz sand particles and boronized coating.

Materials	Density	Elastic Modulus	Poisson’s Ratio
	kg/m^3^	*E*/GPa	*μ*/-
Quartz sand	2650	73.1	0.16
Boronized coating	7150	395	0.20

**Table 2:** Comparison of the exponents in different erosion predictive models for brittle material.

Predictive Models	*e*_1_	*e*_2_	*e*_3_	*e*_4_	*e*_5_	Research Methods
Evans et al. [30] (Erosion volume)	3.2	1.85	1.58	−1.3	−0.25	Theoretical study
Lawn et al. [33] (Erosion volume)	2.4	1.85	1.2	−1.3	0.11	Theoretical study
Hassani et al. [26] (Erosion volume)	2.3	3.13	1.0	−1.47	−2.5	FEM study
This study (Erosion mass)	2.35	1.26	1.0	−1.64	−1.08	Experimental study

## Data Availability

The authors confirm that the data supporting the findings of this study are available within the article and the referenced article.

## Nomenclature

*D*	diameter of a single spherical particle (μm)
*D*_32_	Sauter average diameters of particles (μm)
*E*′	effective elastic modulus, which is defined by Equation (3) (GPa)
*E_M_*	erosive weight-loss of the boronized coating (mg·h^−1^)
*E_p_*	elastic modulus of particles (GPa)
*E_t_*	elastic modulus of coating (GPa)
*E_e_*	elastic potential energy during impact, which is defined by Equation (4) (J)
E_m_	kinetic energy of the particle, which is defined by Equation (5) (J)
*F*	contact force during the normal impact, which is defined by Equation (2) (N)
*H*	micro hardness of the target (GPa)
*P*	impact stress generated during the particle impingement, which is defined by Equation (8) (GPa)
*R_p_*	radius of the incident particle (μm)
K_C_	fracture toughness of coating (MPa·m^1/2)^
*V*	velocity of the incident particle (m·s^−1^)
*d*_p_	diameter of quartz sand particles (μm)
*h*	indentation depth in the impingement process (μm)
*h*_max_	maximum indentation depth during the impingement process (μm)
*l*	initial defect length, which is assumed between 0.1−1 μm (μm)
*m*	mass of the incident spherical particles (g)
*m_p_*	cumulative mass of erodent particles (g)
*m*_1_	mass of target before erosion test (mg)
*m*_2_	mass of target after erosion test (mg)
*q*	mass flow rate of the incoming particles per unit time (kg·h^−1^)

## Greek Symbols

*β*	particle incidence angle (°)
*σ*_crit_	critical stress of the crack propagation at the defects, which is defined by Equation (1) (GPa)
*μ_p_*	Poisson’s ratio of particles *(*−)
*μ_t_*	Poisson’s ratio of coating *(*−)
*ρ_c_*	density of the coating (g·cm^−3^)
*ρ_p_*	density of particles(g·cm^−3^)

## Abbreviations

PIV:	particle image velocimetry
CCD:	charge coupled device
SEM:	scanning electron microscope
BSE:	back-scattered electron
FEM	finite element method
